# Case Report: An unusual case of cardiac anaphylaxis in the maintenance phase of *vespula* venom immunotherapy

**DOI:** 10.3389/falgy.2025.1583909

**Published:** 2025-04-23

**Authors:** Silvia Brunetto, Federica Buta, Sebastiano Gangemi, Luisa Ricciardi

**Affiliations:** Department of Clinical and Experimental Medicine, School and Operative Unit of Allergy and Clinical Immunology, Policlinico “G. Martino”, University of Messina, Messina, Italy

**Keywords:** vespula sting anaphylaxis, VIT, maintenance phase, tryptase, cardiac anaphylaxis

## Abstract

**Background:**

Cardiac involvement in anaphylaxis remains difficult to assess; however, histamine release during an anaphylactic reaction can induce functional and metabolic alterations in the myocardium. Mast cells, identified within myocardial fibers, perivascular tissue, and arterial structures, play a crucial role in systemic and cardiac anaphylaxis through the release of inflammatory mediators, including histamine, platelet-activating factor, cytokines, chemokines, tryptase, chymase, prostaglandins, and leukotrienes. Hymenoptera venom immunotherapy (VIT) is the most effective strategy for preventing systemic reactions in sensitized individuals. Although VIT is generally well tolerated, severe allergic reactions can occur, particularly during the build-up phase, while they are rare in the maintenance phase.

**Case report:**

We present the case of a 57-year-old male with a history of severe systemic reactions (SSR) to Vespula stings who experienced cardiac anaphylaxis during the maintenance phase of VIT. He started VIT with a conventional up-dosing schedule, which was well-tolerated. However, during the third monthly maintenance dose, he developed an anaphylactic syncopal episode with a right bundle branch block (RBBB) on ECG. He was treated promptly with adrenaline, corticosteroids, and antihistamines, and his ECG normalized within 20 days.

**Conclusions:**

This case underscores the potential cardiac involvement in anaphylaxis during VIT maintenance and highlights the need to systematically evaluate cardiovascular manifestations during anaphylaxis episodes to optimize risk assessment and management.

## Introduction

Cardiac involvement in anaphylaxis is difficult to assess, but it has been described that histamine release during an anaphylactic reaction can cause functional and metabolic changes in the heart (1).

Mast cells identified in human heart are involved in systemic and cardiac anaphylaxis through the release of inflammatory mediators such as histamine, platelet activating factor, cytokines, chemokines, tryptase, chymase, prostaglandins and leukotrienes; in the heart they can be found between myocardial fibers, in the perivascular tissue, in the adventitia and arteria intima (2).

Hymenoptera stings can trigger systemic allergic reactions that range from mild symptoms, such as hives, to severe and potentially fatal anaphylaxis in sensitized individuals (3).

The most effective treatment for preventing systemic reactions upon re-sting, including life-threatening episodes, is Hymenoptera venom immunotherapy (VIT) (4).

The schedule for VIT administration requires an induction phase followed by a maintenance phase; during the induction phase the venom is injected subcutaneously at increasing doses until the cumulative dose of 1 ml at the concentration of 100 microgr/ml (mcg/ml) is reached; in the maintenance phase the venom extract is administered at regular intervals to induce and maintain the state of tolerance (5).

VIT is generally a safe treatment; severe allergic reactions have been reported during the build-up phase of treatment while these events are rare during maintenance therapy (6, 7).

We present the case of a 57-year-old male with a history of severe systemic reactions (SSR) to Vespula stings who developed cardiac anaphylaxis during the maintenance phase of VIT.

In the context of our case report, we conducted a systematic review of the available studies to assess the presence of comparable clinical presentations, focusing on cardiac involvement in anaphylaxis.

## Case description

**Figure 1:**
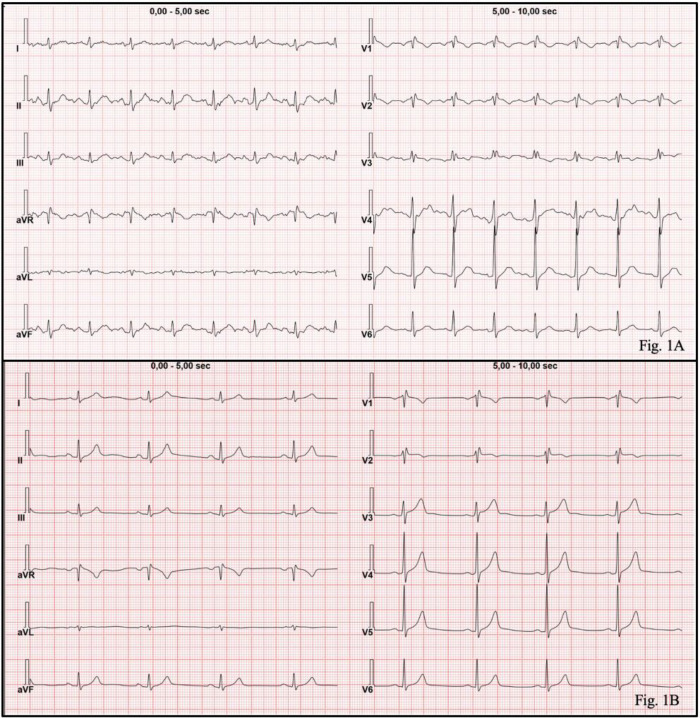
**(A)** The ECG, performed during the anaphylactic reaction after VIT maintenance dose administration, showed a RBBB. **(B)** The ECG performed 20 days after the anaphylactic reaction was normal showing sinus rhythm.

A 57-year-old male with a history of SSR to Hymenoptera stings was referred from the Emergency Department to the Allergy and Clinical Immunology Unit of the University Hospital G. Martino in Messina, Italy, following an anaphylactic episode, characterized by systemic urticaria and syncope, triggered by an Hymenoptera sting. He recovered after treatment with 1 gr of intra venous (i.v.) hydrocortisone, intramuscular (i.m.) chlorpheniramine and 500 mcg i.m. adrenaline. The allergy workup through personal medical history revealed a previous episode, 5 years before, of grade IV anaphylaxis according to Mueller's classification (8), after a wasp sting. At that time the patient had been found unconscious in a public space and urgently transported to the Emergency Department under a red code status. The patient had been advised to undergo an allergic screening to evaluate the feasibility for VIT. However, after recovering from anaphylaxis, he declined further allergy investigations as he could not afford the expenses of VIT. Therefore, he had been instead prescribed an adrenaline auto-injector with instructions on its proper use. After having experienced the second anaphylactic event following a wasp sting, the patient was evaluated in our allergy unit. This time he gave his consent to allergy screening; skin testing with airbone allergens such as dust mites, fungi, pollens, animal dander were negative while skin testing with *Vespula, Honey Bee, Polistes and Vespa crabro* venom extracts (Anallergo, Scarperia and San Piero, Italy) and venom specific IgE measurements (Pharmacia-Cap System, Stockholm, Sweden) confirmed Hymenoptera venom allergy (HVA) due to sensitization to *Vespula* venom. Routine blood tests were within normal limits, total IgE was 7.43 KU/L (normal range <100 KU/L) and Baseline Serum Tryptase (BST) levels were 6.8 ng/ml therefore within the normal range of 1.0–11.4 ng/ml (ImmunoCAP Tryptase Fluoroenzyme-immunoassay, Thermo Fisher/Phadia AB, Uppsala, Sweden). The REMA score calculation was applied (9, 10) (Table 1) using a cut-off ≥2 as a high-risk score for clonal mast cell disorders (CMD). The REMA score was calculated according to gender (+1 for males), presence of urticaria as skin symptom during the SR (−2 if present), syncope during the SSR (+3 if present) and BST levels (−1 for levels <15 ng/ml). Therefore, the REMA score resulted in +1, indicating no immediate suspicion of CMD. The patient agreed to initiate VIT as, in the meantime, a VIT treatment, Alutard *Vespula* (ALK-Abellò, Milan, Italy), had been approved by the European and Italian medicines agencies as a pharmaceutical intervention to be carried out in a hospital setting and supplied in Italy to patients with HVA through the Italian National Health System. The patient commenced VIT with Alutard *Vespula,* a depot *Vespula* venom extract adsorbed on aluminum hydroxide. A conventional up-dosing schedule was followed (Table 2) allowing to reach the maintenance dose of 100.000 standard quality units (SQ-U) equal to 100 mcg of Vespula venom extract in 15 weeks. VIT up-dosing was well-tolerated and successfully completed. The maintenance phase was continued monthly with subcutaneous injections (s.i) of 100.000 SQ-U equal to 100 mcg of *Vespula* venom extract. However, 8 min after the third monthly dose injection, the patient experienced an anaphylactic syncopal episode without any other clinical manifestations such as dyspnea, urticaria, tremors, and nausea. Immediate management of the anaphylactic shock included i.m. administration of 500 mcg of adrenaline followed by 1 g of i.v. hydrocortisone, and 10 mg i.m. chlorpheniramine, along with intravenous fluids. Consciousness was promptly restored, and vital signs were monitored. The ECG (Figure 1A), reviewed online by a cardiologist, showed a right bundle brunch block (RBBB); this sign had never been present in previous ECGs. As cardiac enzymes resulted within normal ranges, no cardiovascular treatment was suggested. Serum tryptase was measured straight after anaphylaxis and it resulted slightly above the normal range (15.6 ng/ml). As the patient recovered completely, after 4 h of clinical observation, he was discharged from hospital.

**Table 1:** The table reports the REMA score developed by the Spanish network on mastocytosis (9, 10), a support tool for determining which patients with previous anaphylaxis require additional diagnostic evaluation.

Variable		Score (a cut-off ≥2 means that the patient is likely to have a clonal mast cell disorder)
Gender	Male	1
	Female	−1
Clinical Symptoms	No urticaria and no angioedema	1
	Urticaria and/or angioedema	−2
	Presyncope or Syncope	3
Baseline serum Tryptase	<15 ng/ml	−1
	>25 ng/ml	2

**Table 2:** The table reports the VIT schedule used, starting with a conventional Up-dosing schedule and followed by monthly maintenance doses every 28 days.

Vial n.	Concentration Sq-U/ml	Week n.	Injection n.	Volume ml	Dosage Sq-U/ml
1	100	1	1	0.2	20
	100	2	2	0.4	40
	100	3	3	0.8	80
2	1,000	4	4	0.2	200
	1,000	5	5	0.4	400
	1,000	6	6	0.8	800
3	10,000	7	7	0.2	2,000
	10,000	8	8	0.4	4,000
	10,000	9	9	0.8	8,000
4	100,000	10	10	0.1	10,000
	100,000	11	11	0.2	20,000
	100,000	12	12	0.4	40,000
	100,000	13	13	0.6	60,000
	100,000	14	14	0.8	80,000
	100,000	15	15	1.0	100.000
4	100,000	Monthly	17	1.0	100.000

Other variables that could have caused a systemic reaction rather than VIT were ruled out as the patient had eaten his usual breakfast 4 h before without taking any drugs.

Twenty days after the event, the patient underwent further cardiology evaluation, and the ECG was normal showing spontaneous normalization (Figure 1B). Another tryptase measurement yielded a value of 11.1 ng/ml.

The above-described severe adverse drug reaction (SAD) has been reported to the Italian medicines' agency with the code number 970329.

## Discussion

Hymenoptera VIT is a well-established, highly effective intervention for preventing systemic reactions in patients with HVA (4); adverse reactions during VIT administration, particularly in the build-up phase, have been reported in 16%–40% of patients treated with *Apis* VIT and in 4%–19% of patients treated with *Vespula* VIT, while severe allergic reactions are rare (11–14). It has been reported that prophylactic administration of Omalizumab could be considered in patients with previous adverse reactions to VIT, but it is still an off label use (6). This case report underscores the complexity of risk stratification before starting VIT, particularly when BST levels are within normal range. Serum tryptase, a marker of mast cell activation, is reported to have a key role when evaluating the risk of anaphylaxis (15). BST has long been regarded as a biomarker for assessing the risk of SSR in subjects with a diagnosis of HVA (16). Elevated BST levels, particularly those above 11.4 ng/ml, are typically associated with a higher risk of severe anaphylaxis (17). Nevertheless, BST levels of 5 ng/ml or 8 ng/ml were present in subjects with SSR after a field sting; in addition, recent studies have shown that tryptase levels traditionally considered as normal may still portend a higher risk of severe reactions in patients with a history of severe HVA, undergoing VIT (18, 19). Our patient's BST levels, though not initially alarming based on traditional thresholds, increased slightly when evaluated during anaphylaxis. This data is supported by previous findings of serum tryptase levels ≥ 8.23 ng/ml identifying anaphylaxis when the blood is drawn less than 6-h from the beginning of anaphylaxis (20). Routine laboratory tests of anaphylaxis are up to now limited to the evaluation of serum total tryptase levels; further research is still needed to be able to evaluate other biomarkers of anaphylaxis such as PAF, chymase, carboxypeptidase A£, dipeptidyl peptidase, basogranulin, and CCL-2; the half-life of these biomarkers is challenging for their routine measurement in real life (21). A multidisciplinary approach should be or is expected to be used to analyze blood samples collected from anaphylaxis patients in the Emergency Department. Previous studies have reported the involvement of cytokines such as IL-6 and IL-10 in such cases (22). Furthermore, biomarkers of anaphylaxis could also be searched in other body fluids such as urine samples (23). A Spanish network on Mastocytosis has developed a score, **t**he so-called REMA score (9, 10), which is an important support tool for determining which patients with previous anaphylaxis require additional diagnostic evaluation (Table 1). The occurrence of a severe anaphylactic episode during the maintenance phase of VIT in our patient, with a high REMA score, underscores the need for a more personalized approach to anaphylaxis risk assessment, particularly in cases where tryptase levels are only slightly elevated (24–26). Patients with a REMA score of less than 2 and a level in the normal range of tryptase may not require immediate investigation. However, those with higher scores (≥2) often need further diagnostic workup, such as bone marrow biopsy and KIT mutation analysis to rule out clonal mast cell disorders (25, 27). In our patient the SR to the maintenance dose of Vespula VIT caused syncope with ECG alterations; the REMA score this time was >2 because of hypotensive anaphylaxis without skin symptoms, a score very suggestive of a MCD. Unfortunately, all these considerations are not sufficient to predict a possible cardiac involvement during anaphylaxis. The heart is a mast-cell rich organ as the presence of such cells has been shown in the endothelium of heart vessels (28). Mastocyte release of histamine in the heart has been reported to strongly influence ventricular function and cardiac rhythm (29) and plays a role in anaphylaxis as human heart mast cells express immunoglobulin E receptors, the so called FcɛRI (27). It has been reported that cardiovascular collapse can be immunologically induced by intracardial histamine release (30)*. In* experimental anaphylaxis with guinea pig hearts, quick and prolonged decrease of coronary blood flow, abrupt increase of heart rate, transient increase in ventricular contractility, arrhythmias such as idioventricular rhythm and conduction defects, were elicited; the latter ranged from partial to complete atrioventricular block (31). Heart involvement in animal models of systemic anaphylaxis has been reported. Guinea pigs were passively sensitized by anti-ovalbumin rabbit serum and 24 h later their hearts were excised and isolated and anaphylactic challenge was induced by a bolus injection of ovalbumin showing possible involvement of platelet activating factor (PAF) in anaphylaxis (32) while mice were sensitized to hazelnut and after repeated oral allergen challenge expansion of cardiac mastcells in the pericardium and myocardium was shown and IL-6 and CCR1/3 and CXCR2 signaling chemokines were significantly elevated (33). No investigation was carried out to verify if the anaphylactic shock after administration of a maintenance dose of Vespula VIT was caused by an IgE mediated mechanism or by other types of adaptive immune responses mediated via IgG or complement as these investigations are not routinely available; in mice IgG subclasses except IgG3 have shown to be capable of inducing anaphylaxis (34). IgG4, instead, are known to be produced during VIT acting as suppressors of anaphylactic responses inhibiting the activation of effector cells (35). Considering other pathways than IgE-dependent in anaphylaxis is an unmet need for a precision medicine approach of the follow-up of patients with anaphylactic reactions, therefore improving risk stratification (36). Anecdotal case reports have been presented describing electrocardiographic alterations during anaphylactic shock such as widespread ST segment elevation, highlighting that the electrocardiogram (ECG) is an essential procedure during an anaphylactic reaction (37, 38). In this report, the RBBB, which developed during anaphylactic shock from VIT, resolved after anaphylaxis treatment, suggesting a direct hemodynamic or inflammatory mechanism in this otherwise healthy patient. To our knowledge, this is the first reported case of cardiac anaphylaxis secondary to VIT administration Likely, this event occurred as a manifestation of Kounis syndrome (KS) which is a consequence of the release of inflammatory cytokines through mast cell activation in the heart, causing cardiac anaphylaxis with coronary artery vasospasms (2), induced by various conditions, drugs, environmental exposures, foods and coronary stents (39).“Type I” KS occurs in patients with normal coronary arteries, in which an allergic reaction triggers mast cell-mediated vasospasm, inducing transient ischemic ECG changes despite normal cardiac enzymes (40). It is thought that this variant could be a manifestation of endothelial dysfunction secondary to endogen oxidative stress induced mastocytes' degranulation (41). Several pharmacologic and environmental triggers have been identified as a cause of KS (42) including food even in the form of exercise-induced-food-dependent KS (43). Clinical cases of KS have been reported after a Hymenoptera sting, both wasp and bee stings, even in children (44–46). Heart involvement in systemic anaphylaxis caused by other types of immunotherapies, non-VIT, has not yet been reported. To explore whether cases of anaphylaxis with cardiac involvement, particularly a RBBB, during the maintenance phase of VIT, had been previously reported a systematic search on PubMed was conducted. Our initial search on PubMed Advanced Search Builder, using the keywords (cardiac anaphylaxis) AND (VIT)) AND (right bundle branch block), made zero results. We then broadened the search to (cardiac anaphylaxis) AND (VIT), which returned eight results but following a detailed analysis, no articles were found to be relevant. We expanded our search to investigate cardiac involvement in anaphylaxis triggered by drugs, foods, and Hymenoptera stings. The initial search recovered 463 articles on cardiac anaphylaxis associated with drug allergy, 259 articles related to food allergy, and 36 articles concerning Hymenoptera venom allergy. However, many of these studies were not directly relevant to our investigation. To improve specificity, we refined the search by applying filters to include only case reports published in English within the last six years, from January 1, 2019, to January 31, 2025. This approach restricted the results to 25 articles on cardiac anaphylaxis correlated to drug allergy, 7 related to food allergy, and 2 associated with Hymenoptera venom allergy. Each article was then meticulously analyzed, and only those presenting well-documented and clinically significant cardiac involvement were included. Case reports in which there was a pre-existing cardiac condition, or the topic was irrelevant or unrelated to our research were excluded. Following this analysis, 4 studies on cardiac anaphylaxis due to drug allergy and 2 studies related to Hymenoptera stings were included, while no articles on food allergy met the inclusion criteria. The selected studies are summarized in Table 3. The results confirmed the exceptional nature of our cardiac anaphylaxis case report from maintenance VIT, as no documented case matched our exact criteria. This finding highlights the need for further research to deepen our understanding of this uncommon presentation and its underlying mechanisms.

**Table 3:** The table reports the articles selected through PubMed research.

Cardiac anaphylaxis and drug allergy				
N.	Author and year	Title	Aims	Conclusions
1	Ebrahimi et al. 2024 (47)	Kounis syndrome type I induced by an intramuscular injection of diclofenac: A literature review based on a case report.	It highlights the potential for nonsteroidal anti-inflammatory drugs (NSAIDs), such as diclofenac, to trigger hypersensitivity reactions with significant cardiac involvement. Specifically, it underscores the importance of recognizing Kounis syndrome, a hypersensitivity-associated acute coronary syndrome, as a possible complication of drug-induced anaphylaxis.	The authors illustrate the rare but critical association between anaphylaxis and acute coronary syndromes, particularly Kounis syndrome type 1, due to coronary vasospasm and highlight the importance of considering hypersensitivity reactions as potential triggers of cardiac events.
2	Morin et al. 2024 (48)	Severe anaphylaxis after chimeric antigen receptor T-cell injection: a case report.	It describes a rare case of severe anaphylaxis with cardiac arrest after Tisagenlecleucel injection for Diffuse Large B cell Lymphoma, who recovered after resuscitation and intensive care treatment, and analyzes data from the FDA Adverse Event Reporting System (FAERS) to assess the occurrence of anaphylactic reactions after CAR-T cell therapy.	The authors conclude that this case and the database analysis indicate that severe anaphylaxis, including cardiac arrest, can occur following CAR-T cell infusion. Clinicians should be aware of this potential complication and be prepared for prompt recognition and management.
3	Gan and Ma 2023 (49)	Hypersensitivity reaction to nedaplatin: A case report and literature review.	It highlights the potential for severe systemic hypersensitivity reactions associated with nedaplatin chemotherapy, emphasizing early detection, prompt intervention, and improved prevention strategies for managing hypersensitivity reactions in patients undergoing nedaplatin treatment.	Systemic hypersensitivity reactions to nedaplatin, though rare, can be rapid and life-threatening. Immediate drug discontinuation and appropriate emergency interventions, including oxygen therapy, corticosteroids, and antihistamines, are crucial for patients’ safety. Close monitoring of infusion rates and vital signs is essential for early detection and prevention.
4	(Erdogan et al. 2022) (50)	An unusual case of concurrent Kounis syndrome and prolonged QT in a young patient.	It emphasizes the rare but critical occurrence of Kounis syndrome and prolonged QTc in a young patient following a single dose of Domperidone and Lansoprazole. The report aspires to raise understanding of the potential cardiovascular risks associated with drug-induced allergic reactions, emphasizing the importance of early diagnosis and careful management to prevent severe complications.	The authors highlighted the importance of considering Kounis syndrome as a possible diagnosis in patients presenting with drug-induced allergic reactions, especially when the symptoms mimic acute coronary syndrome. In this case, the use of Domperidone and Lansoprazole triggered an allergic reaction that led to both Kounis syndrome and prolonged QTc
Cardiac anaphylaxis and food allergy No items included				
Cardiac anaphylaxis and hymenoptera venom allergy				
N.	Author and year	Title	Aims	Conclusions
1	Kawaguchi et al. 2024 (51)	A case of repeated Kounis syndrome after anaphylactic shock: A note for disease management.	It increases understanding of Kounis syndrome as a rare but serious complication of anaphylaxis. It highlights the importance of early recognition, appropriate management, and long-term monitoring to prevent recurrent cardiac events.	The authors underline that Kounis syndrome can manifest simultaneously with or after an anaphylactic reaction and may lead to recurrent coronary events. This case emphasizes the need for vigilant cardiac monitoring following anaphylaxis, as delayed or recurring ischemic episodes can occur, necessitating timely intervention and long-term follow-up.
2	Mondello et al. 2023 (52)	Postmortem Biochemistry and Immunohistochemistry in Anaphylactic Death Due to Hymenoptera Sting: A Forensic Case Report.	This case illustrates the silent lethality of anaphylaxis, the challenges in its forensic diagnosis, and the fatal impact of anaphylaxis with significant cardiac involvement.	The authors concluded that Forensic investigations determined anaphylactic shock due to Hymenoptera stings as the cause of death, with respiratory and cardio-circulatory involvement, including possible coronary vasospasm.

## Conclusions

The clinical case described in this article emphasizes that even during the maintenance phase, generally associated with reduced risk of reactions to VIT, severe reactions can occur.

Even patients with a previous low REMA score but moderately elevated tryptase levels should be carefully monitored, as they may still harbor a latent risk for severe anaphylaxis. A more nuanced and personalized approach to risk stratification, considering all relevant clinical and biochemical markers, is essential to optimize patient safety and prevent life-threatening reactions during long-term VIT treatment.

## Data Availability

The raw data supporting the conclusions of this article will be made available by the authors, without undue reservation.
